# Impact of oncogenic *K-RAS *on YB-1 phosphorylation induced by ionizing radiation

**DOI:** 10.1186/bcr2845

**Published:** 2011-03-10

**Authors:** Mahmoud Toulany, Tim-Andre Schickfluß, Wolfgang Eicheler, Rainer Kehlbach, Birgit Schittek, H Peter Rodemann

**Affiliations:** 1 Division of Radiobiology and Molecular Environmental Research, Department of Radiation Oncology, Eberhard Karls University Tübingen, Roentgenweg 11, D-72076 Tübingen, Germany; 2 Department of Radiotherapy, University of Dresden, Fetcherstrasse 74, D-01307 Dresden, Germany; 3 Department of Diagnostic and Interventional Radiology, University of Tübingen, Liebermeisterstrasse 25, D-72076 Tübingen, Germany; 4 Department of Dermatology, University of Tübingen, Liebermeisterstrasse 25, D-72076 Tübingen, Germany

## Abstract

**Introduction:**

Expression of Y-box binding protein-1 (YB-1) is associated with tumor progression and drug resistance. Phosphorylation of YB-1 at serine residue 102 (S102) in response to growth factors is required for its transcriptional activity and is thought to be regulated by cytoplasmic signaling phosphatidylinositol 3-kinase (PI3K)/Akt and mitogen-activated protein kinase/extracellular signal-regulated kinase (MAPK/ERK) pathways. These pathways can be activated by growth factors and by exposure to ionizing radiation (IR). So far, however, no studies have been conducted on IR-induced YB-1 phosphorylation.

**Methods:**

IR-induced YB-1 phosphorylation in *K-RAS *wild-type (*K-RAS*_wt_) and *K-RAS*-mutated (*K-RAS*_mt_) breast cancer cell lines was investigated. Using pharmacological inhibitors, small interfering RNA (siRNA) and plasmid-based overexpression approaches, we analyzed pathways involved in YB-1 phosphorylation by IR. Using γ-H2AX foci and standard colony formation assays, we investigated the function of YB-1 in repair of IR-induced DNA double-stranded breaks (DNA-DSB) and postirradiation survival was investigated.

**Results:**

The average level of phosphorylation of YB-1 in the breast cancer cell lines SKBr3, MCF-7, HBL100 and MDA-MB-231 was significantly higher than that in normal cells. Exposure to IR and stimulation with erbB1 ligands resulted in phosphorylation of YB-1 in *K-RAS*_wt _SKBr3, MCF-7 and HBL100 cells, which was shown to be K-Ras-independent. In contrast, lack of YB-1 phosphorylation after stimulation with either IR or erbB1 ligands was observed in *K-RAS*_mt _MDA-MB-231 cells. Similarly to MDA-MB-231 cells, YB-1 became constitutively phosphorylated in *K-RAS*_wt _cells following the overexpression of mutated *K-RAS*, and its phosphorylation was not further enhanced by IR. Phosphorylation of YB-1 as a result of irradiation or *K-RAS *mutation was dependent on erbB1 and its downstream pathways, PI3K and MAPK/ERK. In *K-RAS*_mt _cells *K-RAS *siRNA as well as YB-1 siRNA blocked repair of DNA-DSB. Likewise, YB-1 siRNA increased radiation sensitivity.

**Conclusions:**

IR induces YB-1 phosphorylation. YB-1 phosphorylation induced by oncogenic *K-Ras *or IR enhances repair of DNA-DSB and postirradiation survival via erbB1 downstream PI3K/Akt and MAPK/ERK signaling pathways.

## Introduction

The Y-box binding protein-1 (YB-1), which is a member of a family of DNA-binding proteins, is an oncogenic transcription factor that is highly expressed in breast cancers [1,2], colorectal cancer and cancers of the lung, prostate, ovary and bone. Recently, it was shown that YB-1 induces the expression of CD44 and CD49f, leading to enhanced self-renewal and mammosphere growth [3] and resulting in drug resistance [3,4]. In breast cancer, YB-1 was demonstrated to have prognostic and predictive significance through the identification of high-risk patients in the presence or absence of postoperative chemotherapy. Furthermore, the prognostic and predictive significance of YB-1 was found to be independent of tumor biologic factors currently available for clinical decision making [5]. Thus, YB-1 has been proposed as a potent prognostic biomarker for tumor aggressiveness and clinical outcome [6]. The expression of many proto-oncogenes, such as erbB1 [7] and erbB2 [8-10], has been described as being regulated by YB-1. Phosphorylation of YB-1 at serine residue 102 (S102) is required for its function as a transcription factor of erbB1 [7]. As described for basal-like breast cancer cells, the phosphorylation of YB-1 at S102 is carried out by p90 ribosomal S6 kinase [11]. It has been demonstrated that Akt phosphorylates YB-1 at S102 and affects the anchorage-independent growth of breast cancer cells [12]. In line with this effect, it has been shown that YB-1 knockdown induces apoptosis and also decreases phosphorylation of signal transducer and activator of transcription 3 (STAT3), ERK1/2 and mammalian target of rapamycin (mTOR), as well as total mTOR expression [9]. Finally, it has been reported that YB-1 plays pivotal roles in the acquisition of tumor drug resistance through the transcriptional activation of drug resistance genes and genes for growth factor receptors [13,14].

In addition to surgery, radiotherapy is an effective curative approach for many types of cancer, including breast cancer. However, the efficacy of radiotherapy is often challenged by the radioresistance of solid tumors. One of the mechanisms by which tumor cells acquire radioresistance is overexpression or mutational activation of the proteins that regulate survival signaling pathways. In this context, the mutation and overexpression of erbB family members have been well described [15-19].

The erbB family of receptor tyrosine kinases consists of erbB1 (epidermal growth factor receptor (EGFR)), erbB2 (Neu), erbB3 and erbB4. In particular, erbB1 is overexpressed or mutated in many tumors and is associated with a poor outcome of chemo- as well as radiotherapy [18,20-22]. The binding of ligands to the extracellular domain of the receptor induces dimerization, which is necessary for activation of the intracellular receptor tyrosine kinase (RTK) [23]. Moreover, exposure to ionizing radiation (IR) as it occurs during radiotherapy stimulates RTK activity in a ligand-independent manner [24,25]. Both ligand-induced and IR-induced activation of erbB1 mediate the activation of multiple downstream signaling pathways, for example, the phosphatidylinositol 3-kinase (PI3K)/Akt, mitogen-activated protein kinase/extracellular signal-regulated kinase (MAPK/ERK) and Janus kinase (JAK)/STAT3 pathways [26,27]. These intracellular signaling cascades play pivotal roles in regulating growth, proliferation and survival of tumor cells [28]. Most interestingly, the mutation of *K-RAS *has been described as a crucial factor for enhanced activity of the erbB1-dependent PI3K/Akt and MAPK/ERK pathways [25,29,30]. Stimulated Akt has been described as an upstream mediator involved in the activation of YB-1 through phosphorylation at S102 [12]. Because IR is a strong activator of the PI3K/Akt and MAPK/ERK pathways, in the present study we investigated whether IR could induce YB-1 phosphorylation in a panel of breast cancer cell lines. Likewise, the role of YB-1 in the repair of DNA double-stranded breaks (DNA-DSB) and postirradiation survival after exposure to IR was investigated.

Evidence is presented indicating that IR is a strong mediator of YB-1 phosphorylation only in tumor cells with wild-type *K-RAS *(*K-RAS*_wt_); in tumor cells with mutated *K-RAS *(*K-RAS*_mut_), YB-1 is constitutively phosphorylated, and this phosphorylation cannot be further enhanced by exposure to IR. Finally, we found that YB-1 is an important mediator of DNA-DSB repair and postirradiation survival.

## Materials and methods

### Cell lines and reagents

The breast cancer cell lines SKBr3, MCF-7, HBL100 and MDA-MB-231 were used. Additionally, normal human fetal lung fibroblast (HFL), human skin fibroblast cell strains HSF1 and HSF7 and mammary epithelial cell line MCF-10A cells were used. Cancer cell lines and fibroblast cells were cultured in RPMI 1640 and Dulbecco's modified Eagle's medium (DMEM), respectively. Media were routinely supplemented with 10% fetal calf serum (FCS) and 1% penicillin-streptomycin. MCF-10A cells were cultured in endothelial cell basal medium with the addition of medium supplements provided by PromoCell (Heidelberg, Germany) plus 100 ng/ml choleratoxin. Cells were incubated in a humidified atmosphere of 93% air and 7% CO_2 _at 37°C. All experiments were performed in confluent cultures maintained in 10% serum.

Antibodies against phospho-YB-1 (S102) and YB-1, phospho-Akt (S473), phospho-ERK1/2 (T202/Y204) and ERK1/2 were purchased from Cell Signaling Technology (Frankfurt, Germany). Inhibitors against PI3K (LY294002), MEK (PD98059) and anti-K-Ras antibody were purchased from Merck Biosciences (Darmstadt, Germany). Anti-Akt1 antibody was purchased from BD Biosciences (Heidelberg, Germany). Epidermal growth factor (EGF), transforming growth factor α (TGFα), amphiregulin (AREG) and anti-actin antibody were purchased from Sigma-Aldrich (Taufkirchen, Germany). Small interfering RNA (siRNA) against ERK1 and *K-RAS*, as well as a nontargeting siRNA, were purchased from Thermo Scientific (Karlsruhe, Germany).YB-1-siRNA (siRNA-I/II) was purchased from Cell Signaling Technology. Lipofectamine 2000 and Opti-MEM were purchased from Invitrogen (Darmstadt, Germany). Antibody against lamin A/C was purchased from Abcam (Cambridge, UK). The expression plasmids p-EGFP-C1 and p-EGFP/*K-RAS*^V12 ^were described previously [31]. The ErbB1-RTK inhibitors erlotinib and BIBX1382BS, as well as the Akt inhibitor API-59CJ-OH, were described previously [32,33].

### Ligand stimulation, drug treatment and irradiation

For ligand stimulation, cells were treated with EGF, TGFα or and AREG, each at 100 ng/ml, for the indicated time points in each experiment. The ErbB1 inhibitor erlotinib, the PI3K inhibitor LY294002 and the AKT pathway inhibitor (API) were diluted in dimethyl sulfoxide (DMSO), and 10 mM stock solutions were stored at -70°C. The MEK inhibitor PD98059 was prepared as 20 mM stock solution. For treatment, stock solutions were diluted in culture medium, and cells were treated with these solutions to achieve the final concentrations of 5 μM erlotinib, 10 μM LY294002, 20 μM PD98059 and 2.5 μM API-59CJ-OH. Control cultures were treated with medium containing the appropriate concentrations of DMSO. Cells were treated with erlotinib, LY294002 and PD98059 for 2 hours, whereas treatment with API was performed for 72 hours. Irradiation of cells was performed at 37°C. Confluent cells cultured in 10% serum were X-ray-irradiated (100 kVp, 15 mA, 0.3 mm Al additional filtering). The dose rate was 1.7 Gy/minute.

### Protein extraction and western blotting

After undergoing the indicated treatments, cells were washed twice with phosphate-buffered saline and lysed with lysis buffer (50 mM/l Tris·HCl, pH 7.5, 50 mM/l β-glycerophosphate, 150 mM/l NaCl, 10% glycerol, 1% Tween 20, 1 mM/l NaF, 1 mM/l dithiothreitol, protease and phosphatase inhibitors). Following protein quantification using the Bio-RAD *DC *protein assay, samples were subjected to sodium dodecyl sulfate polyacrylamide gel electrophoresis, and assessment of specific proteins in each experiment was performed by Western blot analysis using specific antibodies. After detecting phosphorylated proteins, the blots were stripped and incubated with an antibody against total protein. Densitometry was performed where appropriate using Scion Image software (Scion Corporation, Frederick, Maryland, USA).

### Subcellular fractions

Cytoplasmic and nuclear extracts were prepared according to the instructions contained in the NE-PER Nuclear and Cytoplasmic Extraction Reagent Kit (Pierce Biotechnology, Rockford, IL, USA).

### siRNA transfection

Cells were transfected with 50 nM nontargeting siRNA or specific siRNA using Lipofectamine 2000 transfection reagent according to the protocol of the manufacturer. Twenty-four hours after transfection the media were changed. Cells were used for experiments 4 days after transfection. For knockdown of YB-1, cells were transfected with YB-1 siRNAI/II (Cell Signaling Technology) and for knockdown of K-Ras, a *K-RAS*-specific pool of siRNA (Thermo Fisher Scientific, Bonn, Germany) was used.

### Sequencing of *KRAS*

Total RNA was isolated from frozen cell pellets using the RNeasy mini kit (Qiagen, Hilden, Germany) and reverse transcribed with the Reverse-iT First Strand Synthesis Kit (ABgene, Surrey, UK) using anchored oligo(dT) primers. Exons 1 to 3 of *K-RAS *were amplified from the cDNA using ReddyMix PCR Master Mix (ABGene) with specific primers (sense, GAGAGGCCTGCTGAAAATGA; antisense, TGGTGAATATCTTCAAATGATTTAGT). Amplicons were isolated with QIAquick columns (Qiagen, Hilden, Germany), and both strands were sequenced by a commercial subcontractor (SeqLab, Goettingen, Germany).

### *K-RAS*^V12 ^overexpression

Subconfluent K-RAS_wt _cells (SKBr3 and MCF-7) were trypsinized, and 2 × 10^6 ^cells were transiently transfected with 5 μg of p-EGFP-C1 control vector or p-EGFP/K-RAS^V12 ^by means of electroporation. After 24 hours, the efficiency of transfection was tested by fluorescent microscopy of green fluorescent protein (GFP), and thereafter the media were changed. After an additional 24 hours, cells were used for experiments.

### γ-H2AX foci formation assay

The γ-H2AX foci formation assay was used to evaluate residual DNA-DSB as described previously [34]. Briefly, the cells were cultured on coverglass slides and transfected with 50 nM nontargeting siRNA or specific siRNA against YB-1 and *K-RAS*. After 24 hours, the medium was exchanged with fresh medium. Forty-eight hours later the cells were exposed to single doses of irradiation of 2, 4, and 6 Gy and incubated at 37°C for an additional 24 hours. Thereafter the slides were stained with phospho-H2AX (S139) as described previously. The γ-H2AX foci were counted (70 to 250 cells per treatment condition) and graphed.

### Clonogenic assay

Clonogenic cell survival following radiation exposure was analyzed by means of colony formation assay. Cells were preplated in six-well plates and 24 hours later were mock-irradiated or irradiated with single doses of 1, 1.5, 2, 3 or 4 Gy. Irradiation was performed at 37°C using a Gulmay RS225 X-ray machine (Gulmay limited, Chertsey, UK) with a dose rate of 1.7 Gy/minute and the exposure factors of 150 kVp, 15 mA and 0.3-mm Al additional filtering. To investigate the effect of YB-1 expression on postirradiation survival, cells were transfected with nontargeting siRNA or YB-1-specific siRNA. Three days after transfection cells were preplated in six-well plates, and 24 hours later the cells were mock-irradiated or irradiated with single doses of 1, 1.5, 2, 3 or 4 Gy. In either of the experiments, cultures were incubated for 10 days to allow for colony growth. Colonies of more than 50 cells were scored as survivors. Clonogenic fractions of irradiated cells were normalized to the plating efficiency of nonirradiated controls.

## Results

### Stimulation of YB-1 phosphorylation in breast cancer cells by IR and exposure to erbB1 ligands

**Figure 1:**
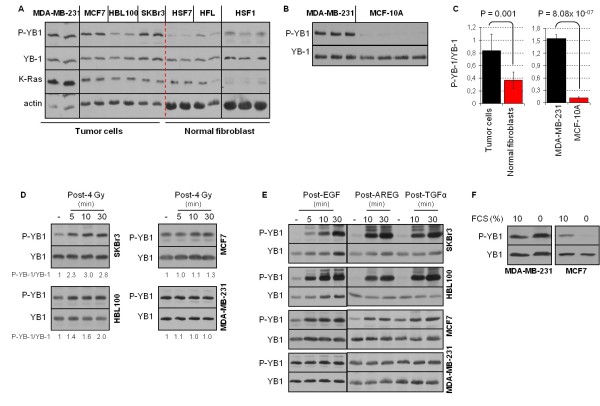
**Phosphorylation of YB-1 stimulated by ionizing radiation and erbB1 ligands**. **(A and B) **Confluent cells (breast cancer cells MDA-MB-231, MCF-7, HBL100 and SKBr3; normal fibroblasts HSF1, HSF7 and human fetal lung fibroblast (HFL); normal mammary epithelial cells MCF-10A) were cultured in 10% serum. Protein samples were isolated from biologically independent cultures, and a sample of 100 μg of protein from each culture was subjected to sodium dodecyl sulfate polyacrylamide gel electrophoresis (SDS-PAGE). P-YB-1, YB-1, K-Ras and actin were detected by Western blot analysis. **(C) **From the densitometric values of P-YB-1 and YB-1, the P-YB-1/YB-1 ratios were calculated for tumor cells versus fibroblasts, as well as normal mammary epithelial cells, and graphed. Statistical analyses were performed using Student's *t*-test. Error bars represent standard deviations (SD). Confluent cells were **(D) **irradiated with 4 Gy of IR or **(E) **treated with 100 ng/ml erbB1 ligand. At the indicated time points after stimulation, protein samples were isolated and subjected to SDS-PAGE. The levels of P-YB-1 and YB-1 were assessed by Western blot analysis. The densitometric values represent the P-YB-1/YB-1 ratio normalized to 1 in nonirradiated controls. **(D) **Phosphorylation of YB-1 after irradiation was tested at least in three independent experiments. **(E) **ErbB1 ligand-induced YB-1 phosphorylation was tested at least in two independent experiments. EGF, epidermal growth factor; AREG, amphiregulin; TGFα, transforming growth factor α. **(F) **Cells (confluent status) were kept in serum-free medium or serum containing 10% fetal calf serum medium. Twenty-four hours after serum depletion samples were isolated, and the level of P-YB-1 was assessed by Western blot analysis. Blots were stripped and incubated with antibody against total YB-1.

The level of basal YB-1 phosphorylation at S102 in a panel of breast cancer cells (MDA-MB-231, MCF-7, HBL100 and SKBr3) was compared to the level of YB-1 phosphorylation in normal cells, that is, human skin and lung fibroblasts (HSF1, HSF7 and HFL) as well as normal mammary epithelial cells (MCF-10A) (Figures 1A and 1B). As shown in Figure 1C, the ratio of P-YB-1/YB-1 is significantly higher in tumor cells than in fibroblasts. The comparisons of the ratio of P-YB-1/YB-1 in tumor cells and normal mammary epithelial cells indicated an even stronger significant difference as tested for MDA-MB-231 and MCF-10A cells (Figures 1B and 1C).

YB-1 has been identified as a direct substrate of Akt [12,35]. As previously reported, IR can activate the Akt ligand independently [30,36]. Therefore, we asked whether IR could induce YB-1 phosphorylation as well. As shown in Figure 1D, IR induces YB-1 phosphorylation differentially. A strong phosphorylation signal was observed in SKBr3, whereas HBL100 showed moderate phosphorylation of YB-1 and phosphorylation in MCF-7 was weak. However, in MDA-MB-231 cells, a lack of IR-induced YB-1 phosphorylation was observed. In this cell line, stimulation with the erbB1 ligand EGF, AREG or TGFα did not induce YB-1 phosphorylation, whereas strong phosphorylation at the indicated times after stimulation was observed in the cell lines SKBr3, HBL100 and MCF-7 (Figure 1D). Although the MCF-7 and HBL100 cell lines have *K-RAS*_wt _status, these cells presented high basal YB-1 phosphorylation. To prove whether the high basal phosphorylation status of YB-1 was due to stimulation by growth factors in the culture medium, P-YB-1 was compared under serum supplementation and serum depletion in MCF-7 cells. As shown in Figure 1F, P-YB-1 was markedly reduced when cells were incubated in serum-free medium for 24 hours. In contrast, serum depletion did not reduce basal YB-1 phosphorylation in *K-RAS*_mt _MDA-MB-231 cells (Figure 1F).

### Constitutive phosphorylation of YB-1 in MDA-MB-231 cells is K-Ras-dependent

**Figure 2:**
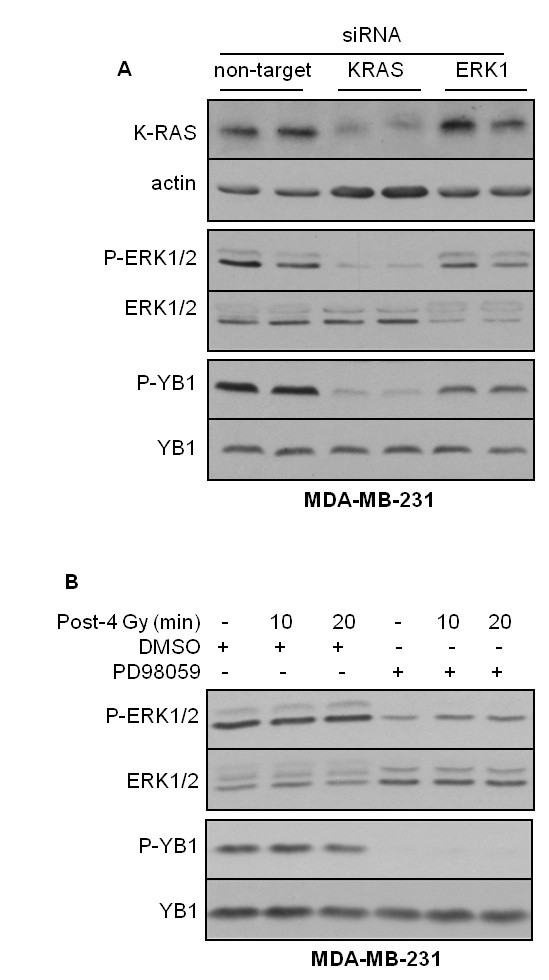
**Constitutive phosphorylation of YB-1 in MDA-MB-231 cells is K-Ras-dependent**. **(A) **Subconfluent cells were transfected with nontargeting small interfering RNA (siRNA) or siRNA against *K-RAS *and extracellular signal-regulated kinase 1 (ERK1) as described in the Materials and methods. Protein samples were isolated, and the levels of K-Ras, actin, P-ERK1/2 and P-YB-1 were detected. The blots were stripped and reincubated with ERK1/2 or YB-1. **(B)** Confluent MDA-MB-231 cells were treated with the MEK inhibitor PD98059 or dimethyl sulfoxide and mock-irradiated or irradiated with 4 Gy ionizing radiation. At the indicated time points after irradiation, protein samples were isolated and P-YB-1 and P-ERK1/2 were detected. The blots were stripped and reincubated with ERK1/2 or YB-1. Representative Western blots of three independent experiments are shown.

MDA-MB-231 cells are characterized by a point mutation at codon 13 in the *K-RAS *gene [37]. This mutation is responsible for the constitutive phosphorylation of ERK1/2 [30]. In addition to ERK1/2 phosphorylation, these cells also present a constitutive phosphorylation of YB-1, which is not further modified after exposure to IR or stimulation with erbB1 ligands (Figures 1D and 1E). Thus, we investigated whether the constitutive phosphorylation of YB-1 in MDA-MB-231 cells is due to the described endogenous expression of mutated *K-RAS *[37]. Therefore, K-Ras expression was downregulated by siRNA, and the level of P-YB-1 was investigated. Using a similar approach, we analyzed the effect of ERK1 on YB-1 phosphorylation downstream of mutated K-Ras. As shown in Figure 2A, *K-RAS *siRNA led to a strong reduction in P-ERK1/2 and P-YB-1 (Figure 2A). Yet, ERK1/2 and YB-1 protein levels were not affected. Likewise, a marked reduction of P-YB-1 was observed when ERK1 was targeted with siRNA. The role of stimulated ERK1/2 phosphorylation on YB-1 phosphorylation was further supported by the results when a MEK inhibitor was used. As shown in Figure 2B, pretreatment of MDA-MB-231 cells with the MEK inhibitor PD98059 markedly blocked YB-1 phosphorylation. Similar to the data shown in Figure 1D, exposure to IR did not induce YB-1 phosphorylation. These results indicates that the constitutive YB-1 phosphorylation in MDA-MB-231 cells is a consequence of mutated K-Ras-mediated ERK1/2 phosphorylation.

### Overexpression of mutated *K-RAS*^V12 ^enhances basal YB-1 phosphorylation

**Figure 3:**
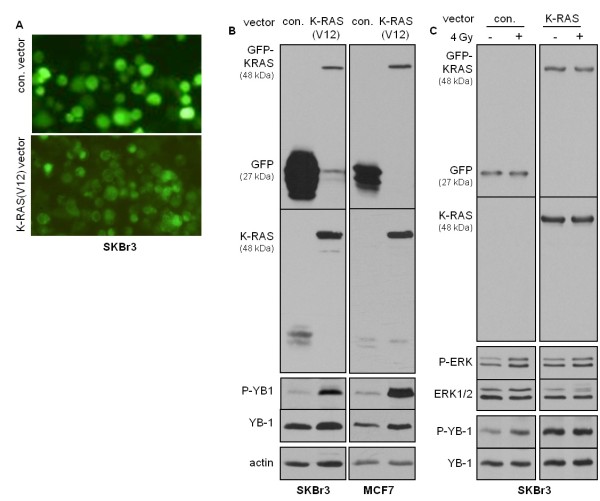
**Overexpression of mutated *K-RAS***^**V12 **^**enhances basal YB-1 phosphorylation**. The indicated cell lines were transiently transfected with pEGFP-C1 control vector (con. vector) or pEGFP/*K-RAS*^V12 ^(*K-RAS*^V12^) as described in Materials and methods. Forty-eight hours after transfection **(A) **green fluorescent protein (GFP) expression was analyzed by fluorescent microscopy and **(B) **protein samples were isolated. The expression levels of GFP and K-Ras were assessed by Western blot analysis. P-YB-1 was detected using the same blots. After the membranes were stripped, each blot was incubated with an antibody against total YB-1. Actin was detected as an additional loading control. The function of *K-RAS*^V12 ^on YB-1 phosphorylation was tested in at least three independent experiments, and a representative Western blot is shown. **(C)** Forty-eight hours after transfecting SKBr3 cells with the pEGFP-C1 control vector or pEGFP/*K-RAS*^V12 ^(*K-RAS*), cells were mock-irradiated or irradiated with 4 Gy ionizing radiation. Ten minutes after irradiation protein samples were isolated. Following sodium dodecyl sulfate polyacrylamide electrophoresis, the expression levels of GFP and K-Ras, as well as the phosphorylation status of ERK1/2 and YB-1, were assessed by Western blot analysis. The blots were stripped and incubated with YB-1 and ERK1/2 antibodies. A representative Western blot of three independent experiments shown.

To investigate the role of K-Ras in the constitutive phosphorylation of YB-1, we further analyzed the status of *K-RAS *in SKBr3, MCF-7 and HBL100 cells. Sequencing of the *K-RAS *gene revealed that none of these cell lines presents a *K-RAS *point mutation in codon 12, codon 13 or 61. To investigate whether mutated *K-RAS*^V12 ^could upregulate YB-1 phosphorylation, we introduced mutated *K-RAS *into *K-RAS*_wt_, SKBr3 and MCF-7 cells. Cells were transiently transfected with either a control pEGFP-C1 vector (indicated as con.-vector) or a vector expressing mutated *K-RAS*, pEGFP-C1/*K-RAS*^V12 ^(indicated as *K-RAS*^V12^). Fluorescence images of living cells transfected with con.-vector and *K-RAS*^V12 ^revealed that GFP in *K-RAS*^V12 ^vector-transfected cells was localized to the plasma membrane, but that in con.-vector-transfected cells it was not (Figure 3A). This is due to posttranslational modification and membrane association of K-Ras (Figure 3A). In con.-vector-transfected cells, GFP expression was not accumulated at the cell membrane, but rather it was equally distributed throughout the cytoplasm. The efficiency of transfection was verified by immunoblotting as well (Figure 3B). In cells transfected with *K-RAS*^V12 ^vector, the expression of K-Ras (21 kDa) resulted in a shift of GFP from 27 kDa to 48 kDa (Figure 3B). The expression of GFP-tagged K-Ras with a molecular weight of 48 kDa was further confirmed by stripping the anti-GFP antibody from the membrane and reincubating the blots with a K-Ras antibody.

In line with our observations of MDA-MB-231 cells, exogenous expression of *K-RAS*^V12 ^in *K-RAS*_wt_, SKBr3 and MCF-7 cells resulted in markedly enhanced basal phosphorylation of YB-1 at S102 (Figure 3B), which prevents further enhancement of phosphorylation by IR (Figure 3C). Thus, these data support the hypothesis that in cells expressing mutated *K-RAS*, the basal phosphorylation of YB-1 is constitutively enhanced and cannot be further stimulated by IR.

### IR-induced YB-1 phosphorylation is mediated by erbB1-dependent PI3K/Akt and MAPK/ERK pathways

**Figure 4:**
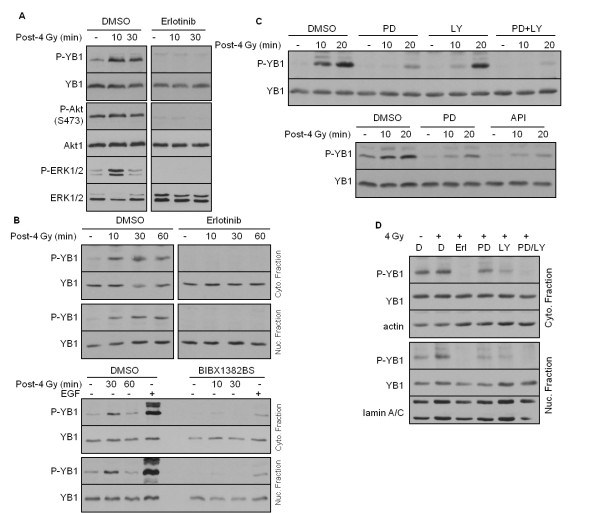
**Radiation-induced phosphorylation of YB-1 is mediated by erbB1-dependent PI3K/Akt and MAP kinase signaling**. **(A) **SKBr3 cells were treated with dimethyl sulfoxide (DMSO) or erlotinib (5 μM) for 2 hours and mock-irradiated or irradiated with 4 Gy IR. At the indicated time points after irradiation, protein samples were isolated and P-YB-1, P-Akt and P-ERK1/2 were detected. The blots were stripped and incubated with antibodies against YB-1, ERK1/2 or Akt1. The effect of erlotinib on IR-induced YB-1 phosphorylation in whole cell extract was tested in two independent experiments. **(B)** SKBr3 cells were treated with DMSO or erlotinib and irradiated as described above. Thereafter 100 μg of isolated cytoplasmic and nuclear fractions were subjected to sodium dodecyl sulfate polyacrylamide electrophoresis. Blots from both fractions were incubated with P-YB-1, followed by stripping and detection of total YB-1. Actin in the cytoplasmic fraction was used as a loading control. The experiment was repeated using the most specific erbB1 tyrosine kinase inhibitor, BIBX1382BS. As a positive control, the 30-minute time point post-epidermal growth factor stimulation was included. **(C and D) **SKBr3 cells were treated with 20 μM PD98059 (PD), 10 μM LY294002 (LY), 2.5 μM API59CJ-OH (API), 5 μM erlotinib (Erl.) or a combination of PD98059 and LY294002 (PD/LY) for 2 hours. Control cells were treated with DMSO. Thereafter cells were irradiated with 4 Gy IR. (C) At the indicated time points and (D) 10 minutes after irradiation, protein samples were isolated and the levels of P-YB-1 were analyzed in (C) whole lysate and (D) cytoplasmic and nuclear fractions. Blots were stripped and reincubated with YB-1 antibody. Actin and lamin A/C were detected as loading controls. The experiments shown in Figures 4C and 4D were repeated at least twice, and representative Western blots are shown.

The phosphorylation of YB-1 at S102 in response to stimulation with EGF has been described as being dependent on p90 ribosomal S6 kinase [11]. In that study [11], Stratford *et al*. showed that the stimulation of SUM149 breast cancer cells with serum, EGF and phorbol 12-myristate 13-acetate (PMA) leads to phosphorylation of YB-1 at S102, which is dependent on the MAP kinase pathway [11]. Because we and others have shown that IR induces activation of erbB1 in a ligand-independent manner [24,25], we tested whether the IR-induced YB-1 phosphorylation shown in Figure 1D could be blocked by erbB1 tyrosine kinase inhibitors. To test this hypothesis, the effect of the erbB1-RTK inhibitor erlotinib on YB-1 phosphorylation was analyzed in whole cell extracts as well as in cytoplasmic and nuclear fractions. Pretreatment of SKBr3 cells with erlotinib resulted in complete inhibition of YB-1 phosphorylation in whole cell extract (Figure 4A) as well as in cytoplasmic and nuclear fractions (Figure 4B). As expected, erlotinib also blocked basal- and radiation-induced P-Akt and P-ERK1/2 in these cells (Figure 4A). To rule out off-target effects of erlotinib, the efficacy of the highly specific erbB1-RTK inhibitor BIBX1382BS [38] on radiation-induced YB-1 phosphorylation was tested in cytoplasmic and nuclear fractions. EGF was included as positive control. As shown at the bottom of Figure 4B, in both cytoplasmic and nuclear protein fractions treatment with BIBX1382BS resulted in a marked reduction of YB-1 phosphorylation stimulated by IR as well as EGF treatment. These data indicate that erbB1-RTK activity is necessary for radiation-induced YB-1 phosphorylation, and this is most likely due to activation of the PI3K/Akt and MAPK/ERK pathways. To test the function of PI3K/Akt and MAPK/ERK pathways in YB-1 phosphorylation, we further investigated whether the inhibitors of PI3K, Akt and MAPK affect YB-1 phosphorylation in irradiated cells. The data shown in Figures 4C and 4D indicate that treatment with either of the inhibitors markedly reduced the phosphorylation of YB-1 at S102. However, optimal inhibition was observed when cells were treated with a combination of PI3K and MEK inhibitors.

### Constitutive YB-1 phosphorylation due to *K-RAS *mutation depends on erbB1 and downstream PI3K/Akt and MAPK/ERK pathways

**Figure 5:**
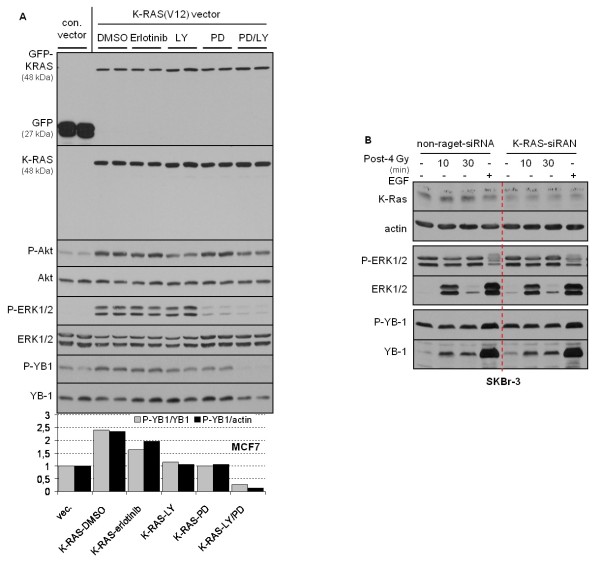
**Constitutive phosphorylation of YB-1 due to *K-RAS *mutation depends on erbB1 signaling**. **(A) **Cells were transiently transfecting with the pEGFP-C1 control vector (con. vector) or pEGFP/*K-RAS*^V12 ^(*K-RAS *^V12^) as described in Materials and methods. Forty-eight hours after transfection, cells were treated with 5 μM erlotinib (Erl.), 10 μM LY294002 (LY), 20 μM PD98059 (PD) or a combination of PD98059 and LY294002 (PD/LY) for 2 hours. Control cells were treated with dimethyl sulfoxide. Protein samples were isolated, and expression levels of green fluorescent protein and *K-RAS*, as well as the phosphorylation status of Akt, ERK1/2 and YB-1, were assessed by Western blot analysis. The blots were stripped and incubated with antibodies against K-Ras, Akt, ERK1/2 and YB-1. The densitometric values represent the ratios of P-YB-1/YB-1 and P-YB-1/actin normalized to 1 in control vector-transfected cells. The effect of indicated inhibitors on *K-RAS*^V12^-induced YB-1 phosphorylation was investigated in at least two independent experiments and representative Western blots are shown. **(B) **SKBr3 cells were transfected with nontargeting small interfering RNA (siRNA) or siRNA against *K-RAS *as described in Materials and methods. Four days after transfection the cells were irradiated with 4 Gy ionizing radiation or treated with 100 ng/ml epidermal growth factor (EGF). At the indicated time points after irradiation and 30 minutes after EGF treatment, protein samples were isolated and subjected to sodium dodecyl sulfate polyacrylamide gel electrophoresis. The levels of K-Ras, P-YB-1 and P-ERK1/2 were detected by Western blot analysis. The blots were stripped and incubated with ERK1/2 and YB-1 antibodies. Actin was used as a loading control. A representative Western blot of two independent experiments is shown.

As IR-induced YB-1 phosphorylation was shown to be dependent on erbB1, PI3K/Akt and MAPK/ERK, we further investigated whether *K-RAS*_mt_-dependent constitutive phosphorylation of YB-1 might be sensitive to the inhibition of erbB1, PI3K and MEK. To this end, *K-RAS*_wt _MCF-7 cells were transiently transfected with con.-vector or *K-RAS*^V12 ^vector, and 48 hours after transfection the cells were treated with the erbB1 inhibitor erlotinib, the PI3K inhibitor LY294002 or the MEK inhibitor PD98059 for 2 hours. Similar to the results shown in Figure 3, overexpression of *K-RAS*^V12 ^resulted in an about 2.5-fold stimulation of YB-1 phosphorylation. Erlotinib reduced mutated *K-RAS *^V12^-induced YB-1 phosphorylation by about 50%, while the PI3K inhibitor and the MEK inhibitor reduced *K-RAS*^V12^-induced YB-1 phosphorylation to the control level. However, the combination of PD98059 and LY294002 (PD/LY) blocked basal and *K-RAS *^V12^-induced YB-1 phosphorylation completely (Figure 5A). These data indicate that phosphorylation of YB-1 due to mutation of *K-RAS *in part depends on activation of erbB1. This is most likely mediated by autocrine production of ligands and is in part independent of erbB1, but it is dependent on activation of the PI3K/Akt and MAPK/ERK pathways.

Because K-Ras strongly induces YB-1 phosphorylation when it is mutated (Figures 3 and 5A), we next analyzed whether phosphorylation of YB-1 in *K-RAS*_wt _cells after irradiation or stimulation with EGF depends on K-Ras expression. Therefore, following downregulation of K-Ras by siRNA, SKBr3 cells were irradiated or stimulated with EGF. As shown in Figure 5B, downregulation of K-Ras did not affect either IR- or EGF-induced YB-1 phosphorylation. A lack of effect of *K-RAS*-siRNA on P-ERK1/2 was observed as well (Figure 5B).

### YB-1 regulates repair of IR-induced DNA-DSB and postirradiation survival

**Figure 6:**
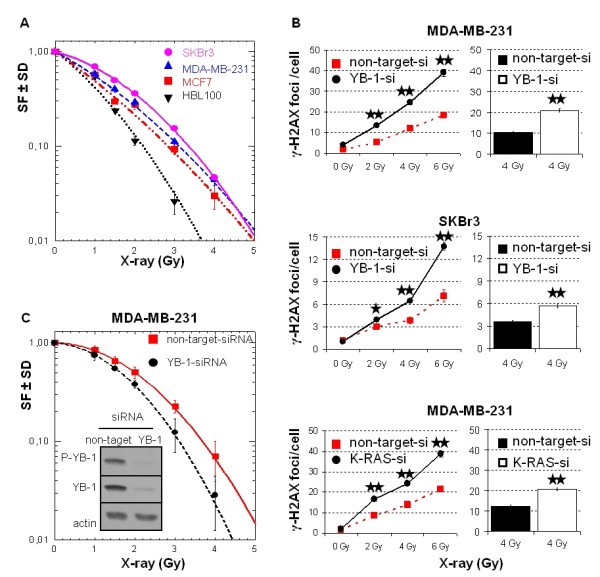
**YB-1 regulates repair of ionizing radiation-induced DNA-DSB and postirradiation survival**. **(A) **Colony-forming assay was performed as described in Materials and methods. Preplated cells were irradiated with single doses of 1, 1.5, 2, 3 and 4 Gy. Ten days later cultures were stained, and colonies with more than 50 cells were counted. The surviving fraction (SF) of irradiated cells was normalized to the plating efficiency of nonirradiated controls. Data represent the average SF ± standard deviation (SD) of at least three biologically independent experiments, with each experiment containing six parallel data sets (*N *= 18). **(B) **Indicated cells were transfected with nontargeting small interfering RNA (siRNA) or siRNA against YB-1 and *K-RAS*. Three days after transfection cells were mock-irradiated or irradiated with 2, 4 or 6 Gy. After 24 hours, the γ-H2AX focus assay was performed. Using a fluorescence microscope, we counted the number of γ-H2AX foci in 70 to 250 nuclei for each individual condition and graphed them. Using Student's *t*-test, we found that YB-1 siRNA as well as *K-RAS *siRNA transfection resulted in significantly enhanced residual γ-H2AX foci (*P < 0.01 and **P ≤ 2.13 × 10^-8^). Bar histograms represent data for residual γ-H2AX foci observed in two independent experiments after irradiation of cells with 4 Gy. **(C) **Three days after transfecting MDA-MB-231 cells with indicated siRNA, cells were seeded into six-well plates for clonogenic assay. Twenty-four hours later cultures were irradiated with indicated doses of ionizing radiation and incubated at 37°C. Ten days later cultures were stained, and colonies with more than 50 cells were counted. The SF of irradiated cells was normalized to the plating efficiency of nonirradiated controls. Data represent the average SF ± SD of six parallel experiments. The significance of the effects YB-1 siRNA on postirradiation survival was assessed using Student's *t*-test. Except for the 1-Gy radiation dose (*P *= 0.089), the effects of YB-1 siRNA at the radiation doses of 1.5, 2, 3, and 4 Gy proved to be statistically significant at the following *P *values: *P*_(1.5 Gy) _= 0.006, *P*_(2 Gy) _= 0.003, *P*_(3 Gy) _= 0.001 and *P*_(4 Gy) _= 0.015. From the cultures used for clonogenic assay, protein samples were isolated and levels of P-YB-1, YB-1 and actin were detected using Western blot analysis.

In addition to its function as a transcription factor, YB-1 is also involved in DNA repair, that is, base excision repair and mismatch repair [39]. In line with this function, it has been demonstrated that YB-1 binds to double-stranded, single-stranded and DNA-containing abasic sites [40]. So far, however, no data demonstrating the function of YB-1 in repair of IR-induced DNA-DSB and postirradiation survival exist. The function of erbB1 and its downstream pathways and the impact of mutated *K-RAS *on repair of DNA-DSB have been demonstrated previously [15,34,41,42]. Therefore, we next asked whether the cells presenting a differential pattern of basal- and radiation-induced YB-1 phosphorylation additionally exert a differential sensitivity to IR. The results obtained by clonogenic assay indicate a differential response in terms of postirradiation survival of the cell lines analyzed. The radiation dose, D_37_, which is required to reduce cell survival to 37%, is 1.95 Gy for SKBr3, 1.65 Gy for MDA-MB-23, 1.35 Gy for MCF-7 and 1.10 Gy for HBL100 cells. We further investigated whether YB-1 activity is involved in the process of DNA-DSB repair and postirradiation survival. For this purpose, a siRNA approach was used. As shown in Figure 6, downregulation of YB-1 by siRNA, either in *K-RAS*_mt _MDA-MB-231 or in *K-RAS*_wt _SKBr3 cells, resulted in impaired repair of DNA-DSB as shown by enhanced residual γ-H2AX foci 24 hours after irradiation. Interestingly, downregulating K-Ras resulted in enhanced frequency of residual DSB to the level observed with YB-1 siRNA. Likewise, siRNA targeting of YB-1 increased radiation sensitivity tested in MDA-MB-231 cells.

## Discussion

This study presents the first evidence that phosphorylation of YB-1 at S102 is induced in tumor cells exposed to IR. Moreover, we provide evidence that oncogenic *K-RAS *due to a mutation in codon 12 or codon 13 leads to constitutive phosphorylation of YB-1.

IR stimulates activation of many cytoplasmic signaling cascades, mostly downstream of membrane-bound receptors [24,43]. ErbB1 is one of the first membrane receptors described that, when overexpressed or mutated, leads to radio- and chemoresistance in a variety of human solid tumors. The expression of erbB1, erbB2 and erbB3 has been reported to be regulated by the transcription factor YB-1 [10,44]. For the nuclear accumulation and induction of transcriptional activity, YB-1 must be phosphorylated at S102 [7]. Phosphorylation of YB-1 at this site under *in vitro *conditions has been described to be dependent on Akt [12,35]. In response to serum, EGF and PMA, the ribosomal S6 kinase (RS6K) has been described as the major enzyme that is responsible for phosphorylation of YB-1 at S102 [11]. Thus, it can be concluded that YB-1 and erbB1 are functionally linked because, on the one hand, YB-1 regulates erbB1 expression and, on the other hand, erbB1 signaling through Akt as well as RS6K stimulates the transcriptional activity of YB-1 through S102 phosphorylation.

It has been well described that IR induces activation of erbB1 and its downstream pathways, mainly PI3K/Akt and MAPK/ERK, in a ligand-independent manner [24,25]. In the present study, we have shown that, as is the case with exposure to erbB1 ligands, IR can induce YB-1 phosphorylation through the activation of erbB1 and the downstream PI3K/Akt and MAPK/ERK signaling cascades. On the basis of these data and the known function of YB-1 in the regulation of erbB1 and erbB2 expression [7,8], it can be assumed that exposure of tumor cells to IR as it occurs during conventional radiotherapy may lead to an enhanced expression of erbB1 and erbB2. Because overexpression of these receptors is associated with radioresistance, YB-1 can thus be proposed as a new candidate to increase the efficacy of molecular targeting strategies in cancer as recently reported [45].

**Figure 7:**
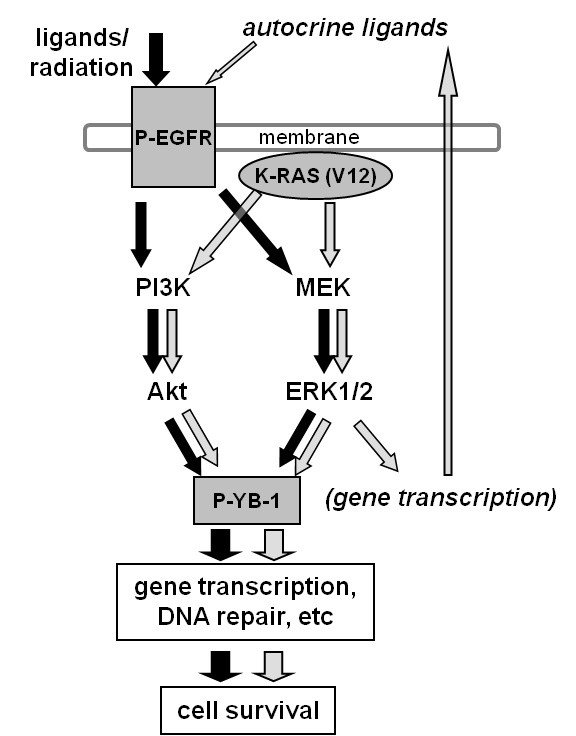
**Importance of K-Ras in regulating YB-1 phosphorylation**. A model illustrating the signaling pathways involved in Y-box binding protein 1 (YB-1) phosphorylation and its function in cell survival after exposure to ionizing radiation and treatment with erbB1 ligands or due to expression of oncogenic *K-RAS*.

The mutation of *K-RAS *is one of the most common genetic alterations in human tumors [46,47]. Oncogenic activation of K-Ras plays a central role in tumor progression and has been associated with resistance to therapy and reduced overall patient survival [48,49]. It has been demonstrated in many cell lines, either with endogenously or exogenously introduced *K-RAS *mutation, that the production of erbB1 ligands, mainly TGFα and AREG, is upregulated [50-54]. Furthermore, K-Ras-mediated autocrine erbB1 signaling through TGFα and AREG contributes to radioresistance [30,55,56]. Here we have shown that endogenously mutated *K-RAS *or overexpression of mutated *K-RAS *in *K-RAS*_wt _cells results in a marked increase in basal phosphorylation of YB-1. Mutated K-Ras due to permanent activation of ERK1/2 results in enhanced autocrine production of erbB1 ligands, such as TGFα and AREG [29,30], which constitutively induce YB-1 phosphorylation (see Figure 1D). In contrast to *K-RAS*_mt _cells, basal phopshorylation of YB-1 in *K-RAS*_wt _cells is sensitive to serum depletion of the culture medium (see Figure 1F), and basal YB-1 phosphorylation in *K-RAS*_wt _cells can be further enhanced by IR or the erbB1 ligands EGF, AREG and TGFα (see Figures 1D and 1E). Thus, our data indicate that YB-1 phosphorylation mediated by *K-RAS *mutation is in part dependent on erbB1 signaling through the PI3K/Akt and MAPK/ERK pathways (see Figure 5). However, downstream pathways of erbB1, such as PI3K/Akt and MAPK/ERK, can also be activated in *K-RAS*-mutated cells independently of erbB1. In this context, mutated K-Ras directly activates the MAPK/ERK pathway [30] through interaction with Raf/MEK and can indirectly activate PI3K/Akt through activating H-RAS [29]. Thus, as summarized in Figure 7, in *K-RAS*-mutated cells, the function of the PI3K/Akt and MAPK/ERK pathways in YB-1 phosphorylation is in part erbB1-independent and directly linked to the activity by K-Ras.

Although growing evidence exists for the function of K-Ras in chemo- and radioresistance, the exact underlying mechanism is not clear. On the basis of recent results, one of the potential mechanisms could be the enhanced repair of DNA-DSB mediated through mutated *K-RAS *[30,42,57]. The data presented in the present study reveal a novel function of mutated K-Ras in regulating YB-1 phosphorylation. Because YB-1 is a multifunctional protein which is also involved in the regulation of DNA repair as described by Gaudreault *et al*. [39] and Hasegava *et al*. [40], phosphorylation of YB-1, either due to *K-RAS *mutation or following irradiation of *K-RAS*_wt _cells, may be necessary for efficient repair of DNA-DSB. The results regarding the γ-H2AX foci support this assumption (see Figure 6). The involvement of YB-1 in DNA-DSB repair is also demonstrated by the fact that YB-1 siRNA, like *K-RAS *siRNA, leads to an enhanced frequency of residual DNA-DSB and affects postirradiation cell survival. The role of YB-1 in the cellular radiation response is further supported by the differential radiation sensitivity of the cell lines tested in the present study. SKBr3 cells, which show marked radiation-induced YB-1 phosphorylation, are the most radioresistant cells, whereas HBL-100 cells, which present the lowest radiation-inducible YB-1 phosphorylation, are the most radiosensitive cells. The radiation sensitivity profile of the four cell lines tested is also in good agreement with the radiation-induced stimulation of YB-1 phosphorylation in these cell lines, which seems to be influenced by the basal phosphorylation status of the YB-1 protein.

## Conclusions

On the basis of the data presented here, it can be concluded that in cells mutated in *K-RAS*, YB-1 is constitutively phosphorylated and this phosphorylation cannot be further enhanced by exposure to IR. However, in *K-RAS*_wt _cells, exposure to IR does induce erbB1 signaling, which mediates YB-1 phosphorylation. As summarized in Figure 7, IR-induced YB-1 phosphorylation in *K-RAS*_wt _or constitutive phosphorylation of YB-1 in *K-RAS*_mt _cells most likely depends on the erbB1 downstream PI3K/Akt and MAPK/ERK pathways, which seem to be responsible for YB-1 phosphorylation and thus the YB-1-mediated repair of DNA-DSB as well as postirradiation survival. Therefore, YB-1 can be discussed as a potential candidate involved in radioresistance of solid tumors, for which targeting of YB-1 could thus be an effective strategy to overcome resistance to radiotherapy.

## Abbreviations

AREG: amphiregulin; DMEM: Dulbecco's modified Eagle's medium; DNA-DSB: DNA double-stranded break; EGF: epidermal growth factor; EGFR: epidermal growth factor receptor; FCS: fetal calf serum; HFL: human fetal lung fibroblast; IR: ionizing radiation; *K-RAS*_mt_: *K-RAS *mutated; *K-RAS*_wt_: *K-RAS *wild type; MAPK/ERK: mitogen-activated protein kinase/extracellular signal-regulated kinase; PI3K: phosphatidylinositol 3-kinase; RS6K: ribosomal S6 kinase; RTK: receptor tyrosine kinase; S102: serine 102; siRNA: small interfering RNA; TGFα: transforming growth factor α; YB-1: Y-box binding protein 1.

## Competing interests

The authors declare that they have no competing interests.

## Authors' contributions

MT drafted the manuscript, designed the experiments and performed the experiments unless stated otherwise. TAS carried out Western blot analysis. WE performed *K-RAS *sequencing. RK was involved in performing the H2AX foci assay. BS provided conceptual suggestions. HPR conceived the studies and was involved in editing the manuscript.
